# Immunomodulatory Effects of *Hedysarum polybotrys* Extract in Mice Macrophages, Splenocytes and Leucopenia

**DOI:** 10.3390/molecules181214862

**Published:** 2013-12-03

**Authors:** Guan-Cheng Huang, Chia-Jung Lee, Kun-Teng Wang, Bor-Chun Weng, Ting-Yi Chien, Sung-Hui Tseng, Ching-Chiung Wang

**Affiliations:** 1 Division of Hemato-Oncology, Department of Internal Medicine, Yuan’s General Hospital, No.162 Cheng Kung 1st Road, Kaohsiung City 80249, Taiwan; E-Mail: guanchenghuang@yahoo.com.tw; 2 Program of Health-Business Administration, School of Nursing, Fooyin University, No.151 Jinxue Road, Kaohsiung City 83102, Taiwan; 3 Institute of Traditional Medicine, School of Medicine, National Yang-Ming University, No.155 Section 2, Linong Street, Taipei City 11221, Taiwan; E-Mail: m303092003@tmu.edu.tw; 4 Department of Education and Research, Taipei City Hospital Renai Branch, No.10, Section 4, Renai Road, Taipei City 10629, Taiwan; 5 School of Pharmacy, College of Pharmacy, Taipei Medical University, 250 Wu-Hsing Street, Taipei City 11031, Taiwan; E-Mails: b8706014@tmu.edu.tw (K.-T.W.); swecon@usc.edu.tw (T.-Y.C.); 6 Department of Microbiology, Immunology and Biopharmaceuticals, College of Life Sciences, National Chiayi University, No.300 Syuefu Road, Chiayi City 60004, Taiwan; E-Mail: brian@mail.ncyu.edu.tw; 7 School of Medicine, College of Medicine, Taipei Medical University, 250 Wu-Hsing Street, Taipei City 11031, Taiwan; E-Mail: m003089010@tmu.edu.tw

**Keywords:** *Hedysarum plybotrys* Handel-Mazzetti (Hedysari Radix), Fabaceae, Astragali Radix, anti-inflammation, splenocyte proliferation, daunoblastina-induced leucopenia mice

## Abstract

Astragali Radix (Huang-Qi) is a popular herbal medicine commonly used as a constituent in tonic herbal preparations. *Hedysarum polybotrys* Handel-Mazzetti is one species used of Astragali Radix. In this study, the immunomodulatory properties of *H. polybotrys* were explored by LPS-activated and SNP-treated RAW 264.7 cells and splenocytes and, daunoblastina-induced leucopenia BALB/c mice. Formononetin was used as the bioactive marker to monitor the quality of the *H. polybotrys* extracts. *H. polybotrys* was extracted with hot-water and methanol, and MeOH extract partitioned with H_2_O (M-H) and ethyl acetate (M-EA) to yield four different fractions. M-EA had the highest formononetin and total proanthocyanidin content and showed stronger inhibitory effects on the production and expression of NO, PGE_2_, iNOS and COX-2 in LPS-activated RAW 264.7 cells and splenocytes than the other fractions. In addition, M-EA significantly stimulated the proliferation of LPS-activated RAW 264.7 cells and splenocytes, enhanced NO radicals scavenging and attenuated NO-induced cytotoxicity. Furthermore, M-EA also significantly increased the rate of recovery of white blood cells level in daunoblastina-induced leucopenia mice. These evidences suggest that this traditional Qi-tonifying herb has potential effects in clinical conditions when immune-enhancing and anti-inflammatory effect is desired.

## 1. Introduction

Astragali Radix (Huang-Qi) is a popular traditional Chinese medicine (TCM) commonly used as a constituent in tonic herbal preparations. According to the record of *Ben-Cao-Gang-Mu* (Compendium of Materia Medica), Astragali Radix is sweet, with warming properties and manifests its therapeutic effects in the spleen and lung meridians [1]. The traditional therapeutic functions of Astragali Radix include reinforcing the flow of Qi in the body, attenuating perspiration, promoting pus discharge, increasing wound healing and induce urine output with resolution of edema [2]. The concept of Qi in TCM is intricate and hard to define. In the study by Yao *et al.*, Qi is defined as the refined nutritious substances constituting the human body and maintaining life activities. In most conditions, Qi is classified as the same as the blood in Western medicine [3]. In Taiwan’s traditional herbal markets, species of Astragali Radix, namely *Astragalus membranceus* (Fischer) Bunge (Huang-Qi) and *Hedysarum polybotrys* Handel-Mazzetti (Hong-Qi) are equally frequently used for similar clinical indications, although their chromatographic characteristics (*i.e*. composition) are different [4].

**Figure 1:**
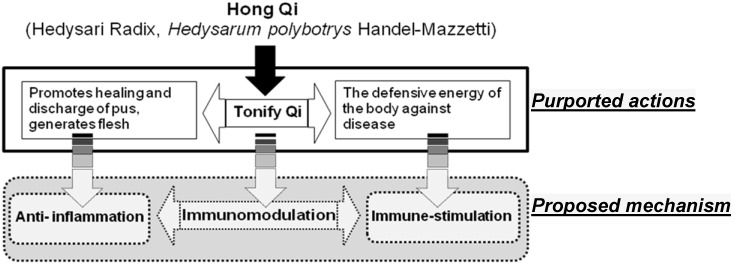
The correlations between traditional and modern pharmacological effects of *H. polybotrys*.

Many herbal medicine extracts or combinations of medicinal plants may have activities on one or more cytokines [5]. Because many cytokines are involved in host responses during both inflammation and immune activation process, herbal medicinal preparations can favorably regulate the whole immune system [6]. Therefore, anti-inflammatory phytomedicines may be a beneficial adjunct to the management of chronic inflammatory disorders due to over-activated immunity [7]. *A*. *membranceus* has been named an immunomodulator, because the extract has been demonstrated to enhance the mitogenic activity of spleen cells and macrophages [8,9]. Anti-inflammatory effects have also been reported for *A*. *membranceus* [10,11,12]. Based on the data from these previous studies, and empirical data from traditional uses, we hypothesize that traditional Qi-tonifying agents may actually possess both immune-modulating and anti-inflammatory activities in modern phytotherapy models (Figure 1). However, little research has discussed these biological activities in *H*. *polybotrys*. Therefore, the aim of this study was to illustrate the pharmacological (Qi-tonifying) activity of *H. polybotrys* with modern scientific methods.

Under inflammatory conditions, macrophages simultaneously produce large amounts of nitric oxide (NO) and superoxide anions (O^−^), which rapidly react to form the peroxynitrite anion (ONOO^−^). ONOO^−^ is a free radical that can interact with lipids, DNA, and proteins and trigger overwhelming cytotoxic cellular responses [13,14]. According to the study by Kim *et al.* [8,11], aqueous-extracts of *A*. *membranceus* enhanced pro-inflammatory cytokines (interleukin (IL)-1β, IL-1α, and IL-6) and mitogenic activity in splenocytes, but inhibited NO production in RAW 264.7 macrophages. Biological activities of herbal materials are related to their constituents. *A*. *membranceus* contains astragalosides and flavonoids. Flavonoids are also the major compounds in *H. polybotrys* [4,15]. Formononetin, an isoflavone, is also particularly rich in *H*. *polybotrys* and has been reported for various bioactivities such as the pro-estrogenic activity [16], inhibition of NO production [17], and ONOO^−^-scavenging effects [18]. Although the main constituents are different between *A*. *membranceus* and *H*. *polybotrys*, these two TCM herbs are used for similar ailments. In this study, we used formononetin as a marker substance for monitoring the qualities of *H*. *polybotrys* extracts. Daunoblastina (daunorubicin) is a potent chemotherapeutic agent. Pharmacological effects of this drug are bound to DNA and inhibited nucleic acid synthesis, while myelosuppression is the most frequently side effects. Aim of this study is to discuss the modulating effects of *H*. *polybotrys* on the production of NO, PGE_2_ and cellular proliferation of RAW 264.7 cells and splenocytes and explore the immunomodulatory effects through daunoblastina-induced leucopenic mice model.

## 2. Results

### 2.1. Total Proanthocyanidin in *H. polybotrys* Extracts

The major components of *H*. *polybotrys* are isoflavonoids. The vanillin assay, which detects flavan-3-ol monomers as well as flavan-3-ol polymers, was used to determine the total proanthocyanidins concentration. As shown in Table 1, the total proanthocyanidin content of the M-EA extract was more than that of the H, M or M-H extracts.

**Table 1:** Quality control and NO inhibitory effects of *Hedysarum polybotrys* extracts and its substance marker, formononetin.

Samples	Quality control				NO inhibition effects	
	Yield (%)	Proanthocyanidin *^a^* (mg/g)	Formononetin *^b^* (mg/g)		IC_50_ of NO^−^ scavenging *^c^*	IC_50_ of NO inhibition in cells *^d^*
H	33.70	2.60	0.013 ± 0.001		0.24 mg/mL	>200 μg/mL
M	15.16	7.68	0.204 ± 0.001		0.50 mg/mL	>200 μg/mL
M-H	14.41	3.75	N.D.		0.18 mg/mL	>200 μg/mL
M-EA	0.75	36.14	5.457 ± 0.174		0.15 mg/mL	55.75 μg/mL
Formononetin	-	-	-		188.28 μM	73.00 μM

Notes: *^a^* Total proanthocyanidin was expressed as mg of catechin equivalents per g of extract; *^b^* Formononetin was a substance marker for *H*. *polybotrys*.; *^c^* Nitric oxide radical was donated by nitroprusside; *^d^* Nitric oxide production from LPS-stimulated RAW 264.7 cells. IC_50_, the 50% inhibitory concentration; H, hot water extract; M, methanol extract; M-H methanol extract followed by water partitioning; M-EA, methanol extract followed by ethyl acetate partitioning.

### 2.2. Concentrations and Bioactivities of Formononetin in *H. polybotrys* Extracts

**Figure 2:**
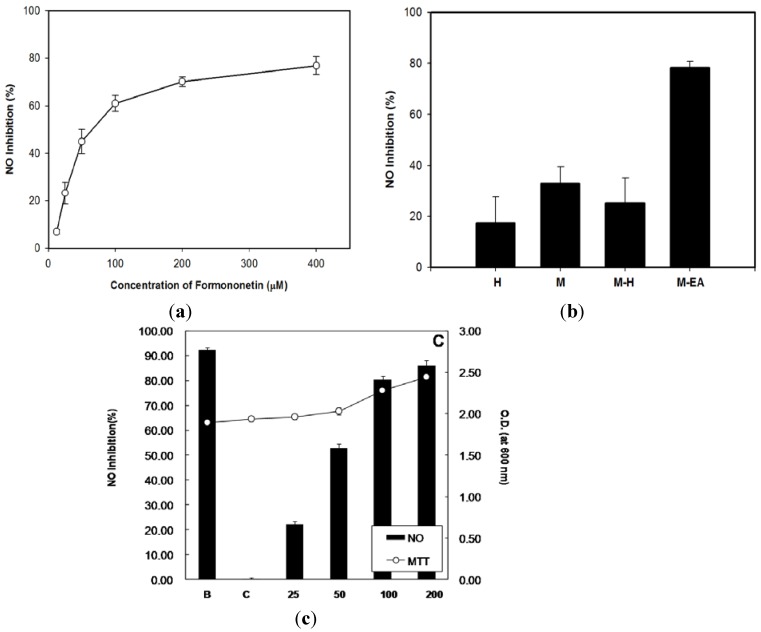
Effects of formononetin and *H. polybotrys* extract on NO production from LPS-activated RAW 264.7 cells. Dose response of formononetin. (**a**) Effects of 4 extract (hot water (H), methanol (M), methanol partitioning with water (M-H), and ethanol partitioning with ethyl acetate (M-EA) at 200 µg/mL. (**b**) Dose response of M-EA on NO production and cell activity. (**c**) The activity of RAW 264.7 cells was measured by an MTT assay (at 600 nm). Values (%) are presented as the mean ± SD, *n* = 3, each with triplicate samples.

**Figure 3:**
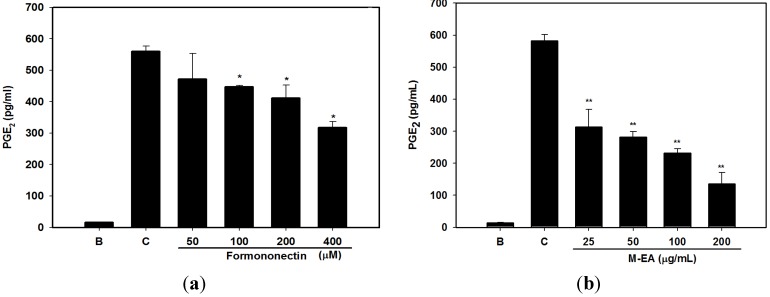
Dose responses of formononetin (**a**) and M-EA (**b**) on PGE_2_ production by LPS-activated RAW 264.7 cells. B: without LPS treatment, C: treatment with LPS and 0.2% DMSO. Values are presented as the mean ± SD, *n* = 3, each with triplicate samples. * *p* < 0.05, ** *p* < 0.005.

**Figure 4:**
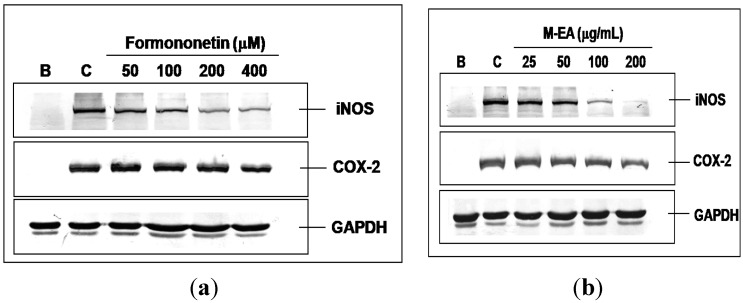
Dose responses orf formononetin (**a**) and M-EA (**b**) on iNOS and COX-2 expressions after LPS-activated RAW 264.7 cells. GAPDH was used as an internal control to identify equal amounts of protein loading in each lane. B: without LPS treatment, C: treatment with LPS and 0.2% DMSO. The results were detected from three separate experiments, from which one picture is shown.

Formononetin is one of the major isoflavone constituents in *H. polybotrys*. Our experiment showed that formononetin inhibited NO and PGE_2_ production in LPS-activated RAW 264.7 cells in a dose-dependent manner. The IC_50_ of formononetin for NO inhibition was 73.0 μM (Figure 2a and Figure 3a, Table 1). Formononetin exerted greater inhibition on iNOS than on COX-2 expression in LPS-activated RAW 264.7 cells (Figure 4a). The above results suggest that formononetin is a potential NO inhibitor.

The M-EA extract contained a higher formononetin level than the other samples (Table 1). The M-EA extracts also showed stronger inhibitory effect on NO and PGE_2_ production in LPS-activated RAW 264.7 cells than that of other extracts (Figure 2b and Figure 3b). In addition, The M-EA extract increased the viability of the RAW 264.7 cells in a dose-dependent manner (Figure 2c). Using western blot assay, we had shown that the M-EA extracts reduced iNOS and COX-2 expressions in LPS-activated RAW 264.7 cells in a dose-dependent manner (Figure 4b).

**Figure 5:**
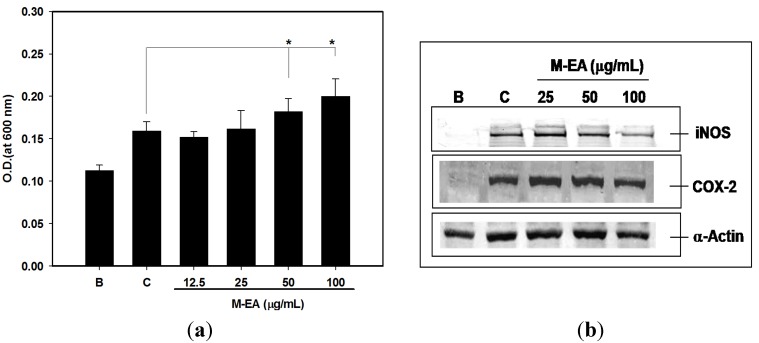
Dose responses of the M-EA on LPS-activated splenocytes. The activity of splenocytes was detected by an MTT assay at 600 nm (**a**). Values are presented as the mean ± SD, *n* = 3, each with triplicate samples. B: without LPS treatment, C: treatment with LPS and 0.1% DMSO. * *p* < 0.05. The iNOS and COX-2 expressions of splenocytes were measured by western blotting assay (**b**). α-Actin was used as an internal control to identify equal amounts of protein loading in each lane. The results were detected from three separate experiments, from which one picture is shown.

Splenocytes was isolated from female BALB/c mice and used as normal cell mixtures. Similarly, treatment with M-EA extract enhanced the viability of LPS-activated splenocytes and inhibited iNOS and COX-2 expressions in a dose-dependent manner, as observed in RAW 264.7 cell experiment (Figure 5).

### 2.3. Attenuation of NO-Induced Cytotoxicity by M-EA Extract

**Figure 6:**
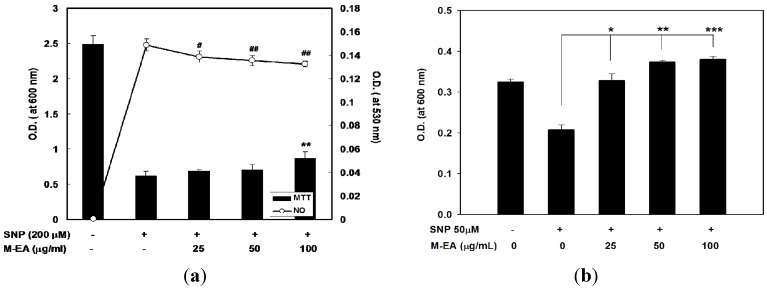
Preventive effects of M-EA on SNP-treated RAW 264.7 cell (**a**) and splenocyte (**b**) death. SNP-induced cytotoxicity was detected by an MTT assay (at 600 nm), and the NO level of the culture medium in RAW 264.7 cells was determined by the Griess reagent (at 530 nm). Values (O.D.) are presented as the mean ± SD, *n* = 3, each with triplicate samples. * *p* < 0.05, ** *p* < 0.005.

SNP is an NO donor and can induce cell death in RAW 264.7 cell and splenocyte at 200 and 50 µM, respectively (Figure 6a,b). When these two cells were cultured with SNP and M-EA, the SNP induced cytotoxicity was attenuated, with concomitant significant reduction of NO concentration in the culture supernatant (Figure 6a,b). In order to verify NO-scavenging activity of formononetin and HP extracts, the compound and the extracts were individually added to SNP solutions *in vitro*. The NO-scavenging activities of formononetin and various extracts were shown in Table 1. M-EA showed the lowest IC_50_ of NO^−^ scavenging levels among all the tested extracts. This result suggested that NO radicals scavenging effect of M-EA could exert protection effects for RAW 264.7 cells and splenocytes against SNP-induced cytotoxicity.

### 2.4. Alleviating Daunoblastina-Induced Leucopenia by M-EA Extract

**Figure 7:**
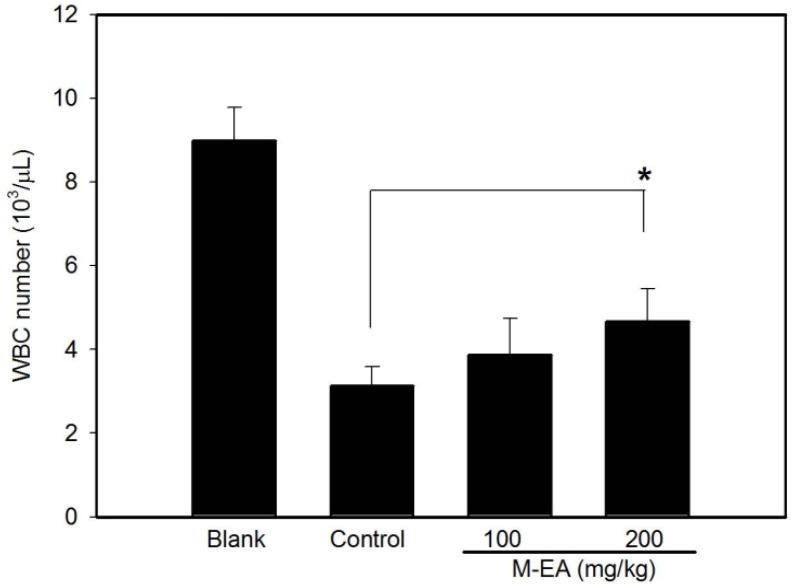
Rise in the WBC level of M-EA treated daunoblastina-induced leucopenic mice. Blank group: The sterile water orally administered and normal saline *i.p.* once. Induced group: The sterile water orally administered and daunoblastina, *i.p.* Test groups: The M-EA at 100 and 200 mg/kg orally administered and daunoblastina *i.p*. Each group contained six mice. * *p* < 0.05.

BALB/c female mice were treated with M-EA (100 and 200 mg/kg) for 6 days and injected intraperitoneally (*i.p.*) with daunoblastina (20 mg/kg) at the 7th day to result in leucopenia in these mice. The WBC level of daunoblastina-induced leucopenic mice was significantly elevated in the M-EA treated-group (Figure 7). The data demonstrated that M-EA could enhance regeneration of immunomudulatory cells in leucopenic mice.

## 3. Discussion

In the present study, we have demonstrated that M-EA fraction of *H. polybotrys* (also known as Hong-Qi in Taiwan) could enhance mitogenic activity and inhibit inflammatory responses in LPS-activated spleen cells and macrophages, while enhanced regeneration of WBC in drug-induced leucopenic mice. In addition, M-EA also could reduce SNP-induced cytotoxicity in RAW 264.7 cells and splenocytes. The results suggest that *H. polybotrys* possesses immune-enhancing and anti-inflammatory effects. The above results supported the hypothesis illustrated in Figure 1, that the traditional qi-tonifying effect of *H. polybotrys* is mediated, at least in part, through immune-enhancing and anti-inflammatory activities.

The human immune system needs to stay in an equilibrated state in order to maintain the health of the host. Weakened or overactivated immune systems may give rise to various disorders ranging from recurrent infections to chronic inflammation [7]. Abnormal cytokine production has been demonstrated in many of these disordered immunity and inflammatory conditions. Herbal medicines usually could modulate the expression of multiple cytokines due to injurious stimuli. Some researchers have proposed that the ability of these herbal preparations to module multiple cytokines may affect the host more favorably then single target therapy does, and thus may offer therapeutic potential for the treatment of many differing disorders involving weakened or overactivated immune system [6]. Among the most frequently studied cytokines, NO, PGE_2_ have pleiotropic activities. PGE_2_ can be produced by many cells of the human body, and deregulated PGE_2_ synthesis have been related to pathological conditions like chronic inflammation, degeneration, or tumor formation [19]. NO released from immune cells such as macrophages play a role in killing tumor cells [20]. However, excessive NO may also lead to cell death. The inhibitory effects of M-EA extract of *H*. *polybotrys* on the production of NO and PGE_2_ and the expression of iNOS and COX-2 in LPS-activated RAW264.7 suggest that the M-EA of *H*. *polybotrys* possesses anti-inflammatory properties. LPS is also a commonly used polyclonal stimulating agent (mitogen), and we have demonstrated that under M-EA treatment, splenocyte and macrophage proliferation in response to LPS stimulation was further augmented, suggesting the therapeutic potential of the extract for the treatment of disorders involving weakened immune response. The underlying mechanism may be due to the NO free radicals scavenging activity of M-EA, since we have demonstrated that the survival of RAW 264.7 cells increased as the concentration of NO decreased in culture medium. The compound that possesses NO scavenging activity inhibited nitrite formation by competing with oxygen to react with NO. This leads to the reduction of nitrite concentration in the assay media. The potential protective effect of M-EA against cytotoxic agents is further supported by the results provided in the SNP-induced cytotoxicity study. NO generated from SNP in aqueous solution at physiological pH reacts with oxygen to form nitrite ions resulting cell death. The active compounds in M-EA inhibited nitrite formation by competing with the oxygen atom to react with NO.

Although our understanding about the medicinal effects of *H. plybotrys* is still far from complete, the M-EA extract of this common herb was found to be enriched in formononetin. In addition, proanthocyanidin components were also reported for the active components in *H. plybotrys* [21,22]. In our HPLC analysis, formononetin was the major components in the M-EA extract of *H. plybotrys*. Evidence has shown that formononetin was able to block the IL-1β-induced NF-κB activation and translocation of NF-κB in INS-1 cell line, indicating the fine anti-inflammatory effects. It’s possible that the immunomodulatory of *H*. *polybotrys* is through this pathway and needed to be explored in the future [23]. Formononetin was reported to activate the expression of estrogen-responsive reporter gene [16]. Formononetin has also been shown to inhibit NO production in LPS-stimulated J774.1 and RAW 264.7 cells, suggesting that this compound may be of therapeutic benefit in various diseases induced by pathological conditions of NO [13,17]. The cardiotoxicity and leukopenia of daunoblastina, a common anti-tumor drug for treatment of leukemia and other neoplasms, was reported to be due to activation of the interior reactive oxygen species. In this study, pretreatment of M-EA could alleviate daunoblastina-induced leucopenia, while the possible mechanism is through the strong free radical-scavenging effects of the proanthocyanidins. After oral administration of M-EA for 7 days, interior radical-scavenging enzymes may be activated and the daunoblastina-induced pro-oxidant effects could be reduced [24,25].

## 4. Experimental

### 4.1. Preparation of *H. polybotrys* Extracts

The dried radix of *H*. *polybotrys* Handel-Mazzetti of the Fabaceae was purchased from an herbal store in Taipei, Taiwan. Assoc. Prof. H.C. Chang (National Laboratories of Food and Drugs, Department of Health, Executive Yuan, Taiwan) authenticated the herbs. Voucher specimens (no. HP-0001) were deposited at the Herbarium of the College of Pharmacy, Taipei Medical University, Taipei, Taiwan.

*H*. *polybotrys* (100 g) was immersed in distilled water (2L), and boiled until half of the original volume was left. The extract was then filtered and freeze-dried to yield hot-water-extracted powder (H). On the other hand, *H*. *polybotrys* (200 g) was refluxed with methanol (2 L MeOH, twice) at 65 °C for 2 h. The combined filtrate was concentrated in a rotary evaporator to remove the methanol to obtain the methanol extract (M). The methanol extract was dissolved in double-distilled water (H_2_O) and then partitioned with ethyl acetate (EtOAc). The aqueous or EtOAc layers were concentrated to dryness on a rotary evaporator to obtain two extract fractions, M-H and M-EA, respectively. Ten milligrams of the four extracts (H, M, M-H, and M-EA) were dissolved in 1 mL of 10% dimethyl sulfoxide (DMSO; Sigma Chemical, St. Louis, MO, USA) and stored at −20 °C until use.

### 4.2. Total Proanthocyanidin Contents Analysis

The vanillin-HCl method was used to determine the proanthocyanidin contents [26]. A 600 µL portion of a freshly prepared solution of vanillin (1 g/100 mL) in 80% H_2_SO_4_ was added to 300 µL of the extract (10 mg/mL). After reacting for 15 min, the absorbance was read at 530 nm. Results were expressed in (+)-catechin equivalents per amount of extracts. Calibration was achieved with aqueous solutions (10~100 µg/mL) of (+)-catechin.

### 4.3. Chromatographic Analysis

An analytical high-performance liquid chromatography (HPLC) system was used to quantify the contents of formononetin in the four extracts. The procedures and instruments of the analytical HPLC system are as described in our previous report [27].

### 4.4. NO Radical-Scavenging Assay

Sodium nitroprusside (SNP) was used as an NO radical (NO^−^) donor [28]. SNP (100 mM) in phosphate-buffered saline (PBS) (pH 7.4) was mixed with different concentrations (125~1000 µg/mL) of the four extracts dissolved in H_2_O and incubated at 37 °C for 90 min [29]. At different intervals, samples (100 µL) of the incubated working solution were removed and mixed with 100 µL of Griess reagent. The absorbance of the reaction solution was measured at 530 nm against the corresponding blank solution. The activity of NO radical scavenging was detected by the spectrophotometric absorbance, and the 50% inhibitory concentration (IC_50_) values of the tested samples were calculated [30].

### 4.5. RAW 264.7 Cell Cultures

RAW 264.7 murine macrophages were obtained from the American Type Culture Collection (Rockville, MD, USA). Cells were cultured in Dulbecco’s modified Eagle’s medium from Sigma (St. Louis, MO, USA) and 10% heat-inactivated fetal bovine serum from Gibco BRL (Grand Island, NY, USA), and then incubated at 37 °C in a humidified incubator containing 5% CO_2_.

### 4.6. Experimental Animals

Five-week-old BALB/c female mice weighing 20~25 g were purchased from the National Laboratory Animal and Research Center (Taipei, Taiwan), and maintained in plastic cages at 21 ± 2 °C with free access to water. They were kept on a 12-h light-dark cycle. Animal care and use protocol was reviewed and approved by the Institutional Animal Care and Use Committee (IACUC, Approval No: LAC-95-0026). The regulations set by IACUC met international laws and regulations.

### 4.7. Preparation of Splenocyte Suspensions

The spleen was isolated from BALB/c female mice and splenocytes were obtained by gentle disruption and filtration through a 40-µm nylon cell strainer (BD, Franklin Lakes, NJ, USA). The erythrocytes were lysed with 0.38% NH_4_Cl-Tris buffer (pH 7.4), while the remaining cells were resuspended in RPMI-1640 with 10 mM Hepes, 10% FBS, 100 mg/L streptomycin, and 100 IU/mL penicillin.

### 4.8. Induction of Inflammatory Response

RAW 264.7 cells (3 × 10^5^ cells/mL) and splenocytes (2 × 10^6^ cells/mL) were treated with 500 ng/mL and 10 µg/mL LPS, respectively, and the test samples were then placed in 6-well plates. After 18 h, levels of nitrite, PGE_2_ iNOS, COX-2 expression and cell viability were measured as described below. The test samples dissolved in DMSO were diluted with the culture medium to concentrations ranging from 25.0 to 3 µM. The final concentration of DMSO was adjusted to 0.05% (v/v).

### 4.9. T Induction of NO-Induced Cytotoxicity

RAW 264.7 cells (3 × 10^5^ cells/mL) and splenocytes (2 × 10^6^ cells/mL) were treated with 500 ng/mL and 10 µg/mL SNP, respectively, and co-cultured with test samples in 96-well plates for 18 h at 37 °C in a humidified atmosphere of 5% CO_2_. The proliferation of RAW 264.7 cells and splenocytes was measured by a MTT assay.

### 4.10. Induction of Leucopenia in Mice

BALB/c female mice were divided into one blank group, one control group, and two experimental groups, with six mice in each group. One hundred and 200 mg of M-EA/kg BW was orally administrated to the two treatment groups, while the blank and control group received sterile water by oral administration once per day for 6 days. After the 6 days of treatment, the control and the experimental groups were injected intraperitoneally (*i.p.*) with 20 mg daunoblastina/kg BW while the blank group received normal saline injection. Whole blood was obtained from the retro-orbital plexus of BaLb/c mice 3 days post induction. The WBC levels of the whole blood were analyzed by an automatic multi-parameter blood cell counter (Sysmex KX-21N, Kobe, Japan). Data are presented as the mean ± standard deviation (SD).

### 4.11. Cell Viability Assay

Mitochondrial respiration, an indicator of cell viability, was assayed by the mitochondrion-dependent reduction of MTT to formazan. Cells in 6-well plates were incubated with MTT (0.25 mg/mL) for 4 h. Cells were solubilized in 0.04 N HCl in isopropanol. The extent of the reduction was measured by the absorbance at 600 nm [30].

### 4.12. Measurement of Nitrite Formation

Nitrate in the medium was converted to nitrite and measured spectrophotometrically by the Griess reaction.

### 4.13. Measurement of Prostaglandin E_2_

The amount of PGE_2_ produced by cells in the media was assessed with a commercially available enzyme immunoassay system (Amersham Pharmacia Biotech, Buckinghamshire, UK), as previously described [31]. Briefly, 50 μL of the supernatant of the culture medium was collected to determine the PGE_2_ level according to the manufacturer’s instructions.

### 4.14. Western Blot Assay

Cells exposed to extracts for 18 h were collected into tubes and then washed with PBS. Protein samples were prepared according to our previous paper [32]. Total protein (25 μg) was used for the Western blot analysis. Proteins were transferred to a nitrocellulose membrane. Membranes were probed using antibodies specific to COX-2, iNOS, GAPDH, and α-actin and visualized using a BCIP/NBT tablet from Sigma Chemical according to the manufacturer’s instructions.

### 4.15. Statistical Analysis

All data are presented as the mean ± SD. Student’s *t*-test was used for comparisons between the treatment and control groups.

## 5. Conclusions

In conclusion, in terms of its anti-inflammatory properties, *H*. *polybotrys* is similar to *A*. *membranceus* although they are two distinct species. *H. polybotrys* is also capable of regenerating immune cell populations under stress. Taken together, the biological activities of *H*. *polybotrys* in macrophage, splenocytes and leucopenia mice suggest the potential usefulness of this traditional Qi-tonifying herb in clinical condition when immune-enhancing and anti-inflammatory effect is desired. Further clinical study of *H. polybotrys* for related diseases and syndromes is warranted.
